# Effects of Pulp and Na-Bentonite Amendments on the Mobility of Trace Elements, Soil Enzymes Activity and Microbial Parameters under *Ex Situ* Aided Phytostabilization

**DOI:** 10.1371/journal.pone.0169688

**Published:** 2017-01-09

**Authors:** Daniel Wasilkowski, Anna Nowak, Grażyna Płaza, Agnieszka Mrozik

**Affiliations:** 1 Department of Biochemistry, Faculty of Biology and Environmental Protection, University of Silesia, Katowice, Poland; 2 August Chełkowski Institute of Physics, University of Silesia, Katowice, Poland; 3 Department of Environmental Microbiology, Institute for Ecology of Industrial Areas, Katowice, Poland; Sun Yat-Sen University, CHINA

## Abstract

The objective of this study was to explore the potential use of pulp (by-product) from coffee processing and Na-bentonite (commercial product) for minimizing the environmental risk of Zn, Pb and Cd in soil collected from a former mine and zinc-lead smelter. The effects of soil amendments on the physicochemical properties of soil, the structural and functional diversity of the soil microbiome as well as soil enzymes were investigated. Moreover, biomass of *Festuca arundinacea* Schreb. (cultivar Asterix) and the uptake of trace elements in plant tissues were studied. The outdoor pot set contained the following soils: control soil (initial), untreated soil (without additives) with grass cultivation and soils treated (with additives) with and without plant development. All of the selected parameters were measured at the beginning of the experiment (t_0_), after 2 months of chemical stabilization (t_2_) and at the end of the aided phytostabilization process (t_14_). The obtained results indicated that both amendments efficiently immobilized the bioavailable fractions of Zn (87–91%) and Cd (70–83%) at t_14_; however, they were characterized by a lower ability to bind Pb (33–50%). Pulp and Na-bentonite drastically increased the activity of dehydrogenase (70- and 12-fold, respectively) at t_14_, while the activities of urease, acid and alkaline phosphatases differed significantly depending on the type of material that was added into the soil. Generally, the activities of these enzymes increased; however, the increase was greater for pulp (3.5-6-fold) than for the Na-bentonite treatment (1.3–2.2-fold) as compared to the control. Soil additives significantly influenced the composition and dynamics of the soil microbial biomass over the experiment. At the end, the contribution of microbial groups could be ordered as follows: gram negative bacteria, fungi, gram positive bacteria, actinomycetes regardless of the type of soil enrichment. Conversely, the shift in the functional diversity of the microorganisms in the treated soils mainly resulted from plant cultivation. Meanwhile, the highest biomass of plants at t_14_ was collected from the soil with Na-bentonite (6.7 g dw^-1^), while it was much lower in a case of pulp treatment (1.43–1.57 g dw^-1^). Moreover, the measurements of the heavy metal concentrations in the plant roots and shoots clearly indicated that the plants mainly accumulated metals in the roots but that the accumulation of individual metals depended on the soil additives. The efficiency of the accumulation of Pb, Cd and Zn by the roots was determined to be 124, 100 and 26% higher in the soil that was enriched with Na-bentonite in comparison with the soil that was amended with pulp, respectively. The values of the soil indices (soil fertility, soil quality and soil alteration) confirmed the better improvement of soil functioning after its enrichment with the pulp than in the presence of Na-bentonite.

## Introduction

The ecotoxicological state of soil has become one of the most serious environmental problems in the world, especially in urban-industrial areas. The contamination of soil with heavy metals (Cd, Cu, Pb, Hg, Ni, Zn) results from mining and smelting, the refinery and chemical industries, industrial and municipal wastes, transport as well as the fertilizers and pesticides that are used in agriculture. Heavy metals are not biodegradable, can accumulate in the food chain and have a negative affect people’s health [1,2]. The potential toxicity of heavy metals depends on the specific form that is present in the soil, their reactivity, mobility, concentration and availability for living organisms. As many studies have shown, the bioavailability of metals constantly changes in the soil and depends on different physicochemical, biological and environmental parameters [2,3].

Due to the high toxicity of heavy metals, there is an urgent need to remove them from contaminated soils or to reduce their migration in the soil profile and thus bioavailability for microorganisms and plants. Strong efforts have been made to develop safer, cheaper and environmentally friendly remediation technologies to replace the invasive physicochemical methods that interfere with the soil structure. The current practice in stakeholder engagement within Europe is mainly focused on the application of “gentle” remediation options (GRO) during land remediation activities [4,5].

The most attractive and promising strategies for reducing the labile fractions of heavy metals in soil seems to be phytostabilization and aided phytostabilization. Phytostabilization aims to restore the vegetation cover of the surface layer of contaminated areas and to promote the *in situ* immobilization of the bioavailable fractions of heavy metals in soil. It uses metal-tolerant plants that can reduce wind erosion, prevent water erosion, immobilize the pollutants by adsorption or accumulation and provide a zone around the roots in which heavy metals can precipitate and stabilize. In turn, aided phytostabilization is a combination of phytostabilization with both organic and inorganic amendments. A number of inorganic additives such as liming materials, phosphate compounds and clay materials are used to immobilize heavy metals, improve soil conditions and facilitate the re-vegetation of contaminated areas [6,7]. Organic materials such as lignite, sewage sludge, compost, peat, ash and tree bark improve the physical nature of soils by increasing the water-holding capacity, creating stable organic heavy metal complexes and decreasing the availability of metals in contaminated soil [7,8]. Increasingly, attention is being paid to industrial waste products including red mud [9] and furnace slag [10], paper mill wastes [11] and biogas residues [12] as potential stabilizers of heavy metals in polluted sites.

Despite the important role of aided phytostabilization in reducing the migration of heavy metals in soil, surprisingly little is known about how various amendments influence per se the microbial biomass, enzymatic activity and community structure of soil. Moreover, the plant-microorganisms-additives interactions still remain poorly investigated. So far, most researchers have focused on the physicochemical analyses of soil contamination and metal accumulation in plant tissues, which has provided very limited information about the chemical behavior and potential impact of metals on the soil microbiome [13–15]. Nevertheless, the success of phytostabilization depends on the inherent nutrient cycling capabilities of the autochthonous microorganisms. Because microorganisms react to changes in their surroundings immediately, any changes in their functional and structural diversity can be an early signal of alterations in the whole ecosystem [1,16]. For this reason, measurements of the microbiological parameters should be combined with the physicochemical analysis of soil properties. The combination of analytical environmental chemistry with microbiological parameters provides more complete and relevant information on the bioavailability of trace elements in soils [1]. Such knowledge is necessary to improve the sustainability and feasibility of phytostabilization.

The importance of any remediation technology, including phytoremediation, is not only removing or reducing the bioavailability of heavy metals, but also the restoration of proper soil quality [17]. According to Alvarenga [11], a variety of bioassays should be considered to evaluate the potential risks to organisms posed by heavy metals. To determine the effectiveness of remediation technology, the implementation of long-term ecological monitoring is indicated. This approach involves systematic measurements of selected parameters and their analysis for a minimum of 10 years in order to regularly control the effect of contamination on the environment and human health [11,18]. An adaptive monitoring was created to improve ecological monitoring [19]. Adaptive monitoring is the only concept that enables monitoring programs to respond to new questions, new information and unexpected events as well as to develop new protocols [20].

The main aim of this study was to evaluate the suitability of organic and inorganic materials (pulp and Na-bentonite) for use as amendments for phytoremediation of Zn, Pb and Cd-contaminated soil and trace elements uptake in *Festuca arundinacea* Schreb. Within the context of an aided phytostabilization the specific goals of this research were to: (1) determine the concentrations of total and bioavailable fractions of Zn, Cd and Pb in soil, (2) assess the plant biomass and heavy metal accumulation in the roots and shoots, (3) evaluate the changes in the microbial biomass and community structure, (4) determine the functional diversity of microorganisms, (5) measure the soil enzymes activity, (6) calculate soil indices and (7) establish the correlations between factors studied.

## Materials and Methods

### Soil and soil additives

Heavy metal-contaminated soil was collected from a directly exposed area of “Orzeł Biały”, a former mine and zinc-lead smelter that is situated between Bytom and Piekary Śląskie, Upper Silesia, Poland (50°36’ N, 18°96’ E). Specific permissions were not required for collection of samples in this location. The work described here did not involve endangered or protected species. The mining (dolomite and coal seam exploitation) and metallurgical activities (Zn, Pb and Cd ore processing) in this area in the years 1846–1981 resulted in the high pollution of the soil with Zn (4693±260.22 mg kg^-1^), Cd (94.50±7.78 mg kg^-1^) and Pb (1420±41.01 mg kg^-1^). Today, the “Orzeł Biały” company is a leader among manufacturers of refined lead and is the largest company in Poland specializing in the recycling of lead-acid batteries. No specific permission was required.

The soil used in the experiment was classified as a silt loam soil with a pH value 6.93±0.1, moisture 11.51±0.29% and organic matter content 4.08±0.06%. The concentrations of CaCl_2_-extractable metals fractions of Zn, Cd and Pb in the soil were 286.02±10.66, 11.32±0.10 and 1.38±0.10 mg kg^-1^ dw, respectively. The other parameters of this soil were reported in a previous study [21].

In this research, two soil amendments were used as potential heavy metals stabilizers–organic pulp from coffee processing (GRANA Sp. z o.o. in Skawina, Poland) (P) and inorganic Na-bentonite (commercially available, e558 C CLAIR T) (SB). The selected parameters that characterize these materials are presented in Table 1.

**Table 1:** Selected physicochemical properties of soil amendments (mean±SD; n = 3).

Material	Moisture (%)	Organic matter (%)	pH	Total concentration			Bioavailable fraction		
				(XRF) (mg kg^-1^ dw)			(0.01M CaCl_2_) (mg kg^-1^ dw)		
				Zn	Pb	Cd	Zn	Pb	Cd
P	82.17±0.52	96.57±0.52	6.28±0.04	5.38±0.20	0.68±0.03	0.26±0.01	1.45±0.30	0.20±0.03	<0.02
SB	10.35±0.44	0.00±0.00	10.41±0.01	<*50.00	<*5.00	<*0.10	<0.02	<0.02	<0.02

P–pulp; SB–sodium bentonite, XRF—X-ray fluorescence spectrometry.

* according to manufacturer data.

### Experimental design

**Fig 1:**
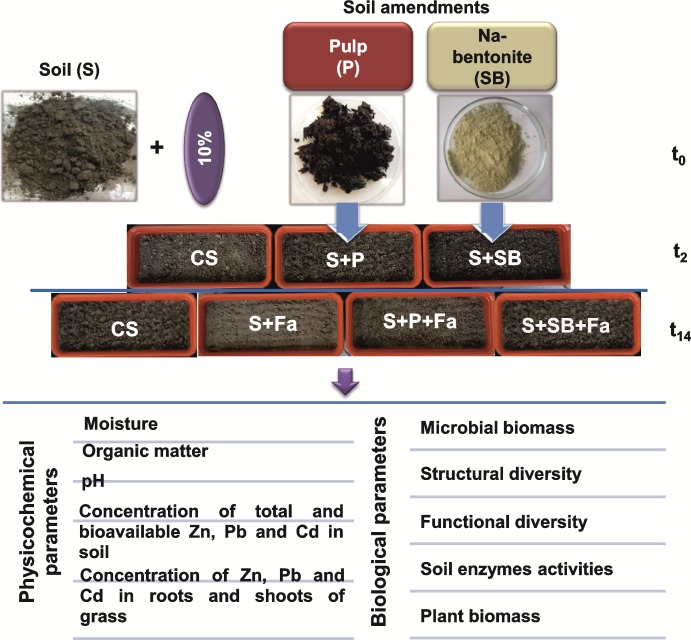
The scheme of experimental design.

The collected topsoil (0–35 cm) was air-dried, passed through a 1-mm mesh screen, weighted and mixed separately with P and SB at a concentration of 10%. This concentration was established as the optimal concentration in the immobilization of heavy metals in the tested soils in a preliminary study in a lab-scale experiment (data not shown). The treated soils, which are described as the S+P and the S+SB, as well as the control soil without any amendments (CS) were transferred to experimental pots (6 kg per each pot) with a depth of 30 cm and an area of 0.07 m^2^. The treated soils were prepared in three replicates for each treatment (a total of 6 pots); however, the control soil was repeated 6 times. Next, all of the pots were placed in an open area of the Institute of Ecology of Industrial Areas (Katowice, Poland) and exposed to an annual cycle of atmospheric conditions (Table 2). After 6 weeks of chemical stabilization, the seeds of a tall fescue (*Festuca arundinacea* Schreb. cultivar Asterix) were seeded (1.75g per each pot) in the enriched soils (S+P+Fa and S+SB+Fa) and in 3 untreated pots (S+Fa). The other 3 pots with the CS (without amendments and vegetation) were used as the control pots (Fig 1).

**Table 2:** Meteorological conditions during the experiment (according to The Institute of Meteorology and Water Management, Poland).

Season	Year	Air temperature (°C)	Rainfall (mm)	Insolation (h)
spring	2014	10	200	540
summer		17	300	725
autumn		10	140	360
winter	2014/2015	1	210	180
spring	2015	9	120	560
summer		22	210	775

The outdoor pot experiment was conducted for 14 months, from May 2014 to July 2015. At the beginning (time t_0_), after 2 months (time t_2_) and at the end of the experiment (time t_14_), soil samples were taken from all of the pots and immediately transported to the laboratory in sterile bags, sieved through a 2 mm sieve and stored at 4°C in polyethylene bags. The soil samples were used to conduct the physicochemical and biological analyses of the soil (Fig 1).

### Soil physicochemical parameters

The pH was measured using the electrometric method in a soil suspension (in H_2_O, 1:2.5, m/v) using a glass electrode after shaking the sample for 60 min (130 rpm) at 20°C (Polish norm PN-ISO 10390:1999). The soil moisture was determined by drying a soil sample (5 g) at 105±5°C to achieve a constant weight [22]. The organic matter content was estimated using the loss on ignition method. For this purpose, a dried soil sample (105°C) was ignited at 550±25°C until a mass constancy was achieved. The soil organic matter content was calculated from the mass difference before and after heating [22]. To determine the total heavy metal concentration, X-ray fluorescence spectrometry (XRF) using a ZSX Primus II (Rigaku) was used. An air-dried soil sample (for 3 days) was pressed in a hydraulic press and analyzed using two crystals, LiF 200 and LiF 220, to determine the concentrations of Zn, Cd and Pb. To measure the bioavailable fractions of metals, a soil sample was treated with 50 mL of 0.01M CaCl_2_ for 5 h (Polish norm ISO 17402:2008(E)). The concentrations of Pb, Zn and Cd in a soil sample were estimated using flame atomic absorption spectrophotometry (AAS ICE 3000, Thermo Scientific).

### Plant biomass and heavy metal accumulation

To determine the dry weight of shoot biomass of the upper ground part of the *F*. *arundinacea* at the end of the experiment (t_14_), freshly mown grass cultivated in the S+P, S+SB and S+Fa was dried at 70±5°C for 72 h until mass constancy was achieved. Prior to the measurements of heavy metal concentrations, the soil adhering to the roots was removed with deionized water. Next, 0.2 g of dried biomass (roots and shoots) was mineralized in a mixture of HNO_3_/30% H_2_O_2_ (1:4, v/v; 9 mL) and analyzed using ASA equipment [23].

### Microbial biomass and community structure

Soil microbial biomass and changes in the microbial community structure were assessed at times t_0,_ t_2_ and t_14_ using the phospholipid fatty acid (PLFAs) method. Total lipids were extracted directly from a soil sample according to the procedure of Frostegård et al. [24]. The lipid material was extracted from fresh soil (2 g) in a one-phase extraction mixture chloroform:methanol:citrate buffer (1:2:0.8, v/v/v; 9.1 mL). The obtained extract was fractionated using a solid phase extraction silicic gel column (Variam, Bond Elut-SI, 500 mg, 3 mL, Supelco^TM^), which was activated with chloroform (5 mL). Neutral lipids were extracted with chloroform (4 mL), glycolipids with acetone (8 mL) and phospholipids with methanol (3 mL). Eluted phospholipids were subjected to a mild alkaline methanolysis with methanol:toluene (1:1, v/v; 1 mL) and 0.2 M KOH solution (1 mL) and incubated in a water bath (15 min, 37°C). Next, a mixture of hexane-chloroform (4:1, v/v; 2 mL), 1 M acetic acid (0.3 mL) were added. The resulting fatty ester methyl esters (FAMEs) were separated, quantified and identified using gas chromatography-flame ionization detection (Agilent 7820A GC-FID) with an Ultra 2-HP capillary column (cross-linked 5% phenyl methyl silicone, 25 m, 0.22 mm id, film thickness: 0.33 μm) and hydrogen as the carrier gas. Qualitative and quantitative FAME analysis was conducted using MIDI Sherlock Microbial Identification System software (MIDI Inc., Newark, USA) and the TSBA library version 6.2B. Calibration of GC was performed using a standard solution No. 1200-A, MIDI Inc. To quantify the concentration of each FAME, the internal standard 19:0 was used (FLUKA, no. 74208-1G).

The identified fatty acids 15:0 *iso*, 15:0 *anteiso*, 15:0, 16:0 *iso*, 16:1 ω7*c*, 16:1 ω5*c*, 17:0 *iso*, 17:0 *anteiso*, 17:0 *cy*, 18:1 ω7*c*, 18:0 10Me and 19:0 *cy* are considered to be of a bacterial origin and they were used as the biomarkers to calculate the bacterial biomass [25]. The fatty acids 18:2 ω6,9*c* and 18:1 ω9*c* were used as indicators of fungi [26]. The 14:0 *iso*, 15:0 *iso*, 15:0 *anteiso*, 16:0 *iso*, 17:0 *iso*, 17:0 *anteiso*, 18:0 *iso* and 18:0 *anteiso* were assigned to Gram-positive bacteria (G+), while Gram-negative bacteria (G-) were characterized by 17:0 *cy*, 19:0 *cy*, 16:1 3OH, 18:1 3OH, 16:1 ω5*c*, 16:1 ω7*c*, 16:1 ω9*c*, 18:1 ω5*c* and 18:1 ω7*c*. The biomarkers for actinomycetes included 16:0 10Me, 17:0 10Me and 18:0 10Me [24]. The sum of all of the FAMEs was used to estimate the total microbial biomass and Shannon’s diversity index according to Eq 1:

$$H^{\prime }=-\sum_{i=1}^{N}p_{i}\;\ln\;p_{i}$$ (1)

where p_i_ means a single PLFA. The biomass of all of the microbiological groups was expressed as nmol g^-1^ dw of soil.

### Physiological level of microbial community

The community level physiological profiles (CLPPs) in a soil sample were obtained using Biolog^®^ 96-well plates EcoPlates^TM^ (BIOLOG Inc., Hayward, USA). This method provides an indication of the potential functional diversity of bacterial communities via the utilization patterns of 31 sole carbon sources in three replicate sets [27]. Violet tetrazolium redox dye is used as an indicator of substrate utilization. Briefly, a 10^−1^ fresh soil dilution in sterile 0.85% NaCl was shaken for 30 min (130 rpm) at 23°C. Next, 100 μL of each sample were inoculated into each well of the EcoPlates^TM^ and incubated at 23°C for 96 h [28]. The rate of the utilization of the substrates was read every 24 h at 590 nm using a MicroStation^TM^ reader. The data were collected using Microlog 4.01 software. Shannon’s diversity index (Eq 1) and species richness (S, the number of substrates utilized with their Omnilog Unit value OU≥100) were also calculated [16].

### Soil enzymes activity

The soil activity in all of the tested soils was assayed via the quantification of selected soil enzymes at t_0,_ t_2_ and t_14_. Dehydrogenases (EC 1.1.1) were used as an indicator of viable microbial cells. The activity of these enzymes was measured according to Friedel et al. [29]. Briefly, 1 mL of 3% 2,3,5-triphenyltetrazolium chloride (TTC) was added to 3 g of soil mixed with CaCO_3_ (10 mg g^-1^) and incubated in the dark for 24 h at 23°C. Next, 1,3,5-triphenyltetrazolium formazan (TPF) as the product of TTC reduction was extracted with ethanol for 2 h and centrifuged (2500 rpm, 5 min), after which the sample was measured colorimetrically at 485 nm.

Phosphatases are commonly used to assess the mineralization of the organic phosphorus. The activities of acid phosphatase (EC 3.1.3.2) and alkaline phosphatase (EC 3.1.3.1) were determined using the protocol designed by Tabatabai and Bremner [30]. The activities of monophosphatases were measured by incubating a soil sample (1 g) with 1 mL of *p*-nitrophenyl phosphate disodium (PNPP) as a substrate in a borate buffer (MUB; Tris, maleic acid, citric acid and boric acid). MUB pH 6.5 and MUB pH 11.0 were used to measure the activity of acid and alkaline phosphatases, respectively. After 2 h of incubation at 23°C 0.5 M CaCl_2_ (1 mL) and 0.5 M NaOH (4 mL) were added to the sample and next the mixture was centrifuged (2500 rpm, 5 min). The absorbance of the reaction product *p*-nitrophenol (PNP) was determined spectrophotometrically at 630 nm.

Ureases (EC 3.5.1.5) belong to the hydrolytic enzymes that catalyze the hydrolysis of urea into carbon dioxide and ammonia. The urease activity was assessed using the method described by Alef and Nannipieri [31] using fresh soil (10 g) and a 10% solution of urea (10 mL) and 50 mM Tris-HCl (pH 9.0) (20 mL) as a buffer. After 3 h of incubation at 23°C, the mixture was centrifuged (2500 rpm, 5 min). Next, sodium phenolate (4 mL) and sodium hypochlorite (3 mL) were added to the supernatant. The ammonia was estimated using a spectrophotometer with a wavelength of 630 nm. All of the analyses were conducted in triplicate for each soil sample. The activities of phosphatases and urease were expressed as μg g^-1^ h^-1^, while the activity of dehydrogenases was expressed as μg g^-1^ 24 h^-1^.

### Soil indices

Based on enzymatic activity and carbon content, a potential biochemical soil fertility index was computed from the Eq 2 [32]:

$${\text{M}}_{\text{W}}=(\text{U}+\text{Deh}+\text{Pal}+\text{Pac})∙\%\text{C}$$ (2)

correlating urease (U), dehydrogenase (Deh), alkaline phosphatase (Pal) and acid phosphatase (Pac) with the % of organic matter (OM) instead of % of C. The equation was finally normalized to get a maximum SQI of one.

The soil quality index (SQI) was selected to evaluate the effectiveness of soil remediation [33]. Among the high-correlated variables (correlation coefficient >0.7, p≤0.05), the concentrations of bioavailable Zn and Cd, water content, bacterial and fungal biomass, as well as the activities of acid and alkaline phosphatases were selected for the SQI described by the Eq 3:

$$SQI=\frac{1}{n}\sum_{i=1}^{n}W_{i}∙S_{i}$$ (3)

where W is the PCA weighing factor and S is the indicator score. The equation was finally normalized to get a maximum SQI of one.

The soil alteration index (SAI) was calculated as the raw canonical coefficients (a_i_) of the PLFA data and the concentration of a specific PLFA (PLFA_i_) [34]. The index was calculated from Eq 4:

$$SAI=\sum_{i=1}a_{i}∙{PLFA}_{i}$$ (4)

The specific PLFAs included: 15:0 *iso*, 16:0 *iso*, 16:0 10Me, 17:0 *iso*, 17:0 *anteiso*, 17:0 *cy*, 18:0 10Me, 16:1 ω5*c*, 16:1 ω7*c*/16:1 ω6*c*, 18:1 ω7*c*, 18:1 ω9*c* and 18:2 ω6,9*c*.

### Statistical analyses

Statistical differences between the soils at a given sampling time were analyzed using a one-way analysis of variance (ANOVA) and were followed by the separation of the treatments from the control, as well as among themselves, by applying the post-hoc LSD (p≤0.05) test. To find any differences in PLFA profiles between the tested soils, principal component analysis (PCA) with the relative abundances of all PLFA markers was performed. All of the statistical analyses were performed using Statistica^®^ 12.5 PL (StatSoft^®^ Inc., USA) and Microsoft Office Excel 2010. For all of the analyses, data were represented as the mean ± the standard deviation (SD) of three replicates.

## Results

### Effects of additives on physicochemical properties of the soils

The effect of pulp (P) and Na-bentonite (SB) on moisture, pH, organic matter content and stabilization of heavy metals in the control, untreated and treated soils at the beginning (t_0_), after 2 months (t_2_) and 14 months (t_14_) of the pot experiment is presented in Table 3.

**Table 3:** Physicochemical properties of soils at t_0_, t_2_ and t_14_ of the experiment (mean±SD; n = 3).

Sampling time	Treatments	Moisture	Organic matter	pH	CaCl_2_-extractable (mg kg^-1^ dw)		
					Zn	Pb	Cd
		(%)	(%)				
t_0_	CS	11.51±0.29^**a**^	4.08±0.06^**a**^	6.93±0.10^**a**^	286.02±0.93^**a**^	1.38±0.10^**a**^	11.32±0.10^**a**^
	S+P	15.58±0.16^**c**^	6.57±0.07^**b**^	7.18±0.01^**b**^	287.30±1.37^**b**^	1.35±0.01^**a**^	10.59±0.20^**b**^
	S+SB	7.01±0.50^**a,b**^	3,94±0.03^**c**^	8.69±0.06^**c**^	201.43±12.37^**c**^	1.00±0.13^**a**^	10.19±0.15^**b**^
t_2_	CS	22.33±0.23^**a**^	3.88±0.01^**a**^	7.22±0.10^**a**^	292.23±6.88^**a**^	1.63±0.11^**a**^	12.21±0.14^**a**^
	S+P	27.27±0.12^**b**^	6.22±0.06^**b**^	7.56±0.06^**b**^	36.19±0.75^**b**^	1.20±0.12^**b**^	4.63±0.26^**b**^
	S+SB	23.15±0.21^**a**^	4.08±0.14^**c**^	8.40±0.05^**c**^	23.68±4.01^**c**^	0.34±0.06^**c**^	3.28±0.17^**c**^
t_14_	CS	4.46±0.01^**a**^	3.78±0.00^**a**^	7.32±0.05^**a**^	265.69±7.34^**a**^	1.92±0.13^**a**^	12.45±0.13^**a**^
	S+Fa	5.42±0.31^**a**^	4.04±0.32^**a**^	6.66±0.01^**b**^	278.52±21.83^**a**^	1.59±0.06^**a,c**^	11.17±0.25^**b**^
	S+P+Fa	24.59±0.02^**b**^	5.73±0.11^**b**^	7.68±0.01^**c**^	25.40±0.45^**b**^	1.29±0.13^**b,c**^	3.13±0.12^**c**^
	S+SB+Fa	33.15±0.03^**c**^	4.18±0.02^**a**^	8.00±0.15^**d**^	25.76±2.11^**b**^	0.97±0.15^**b**^	1.77±0.10^**d**^

CS–control soil; S+Fa–untreated soil with grass; S+P–soil with pulp; S+P+Fa–soil with pulp and grass; S+SB—soil with Na-bentonite; S+SB+Fa—soil with Na-bentonite and grass. Means with the same letter(s) are not significant at p <0,05 within each parameter between the treated and control soils at t_0_, t_2_ and t_14_.

Significant differences (ANOVA, LSD, p≤0.05) in the analyzed parameters were observed between the control, untreated and treated soils (Table 3). At t_0_ the highest content of water (15.58%) and organic matter (6.57%) were measured in the soil that was enriched with pulp (S+P), while the highest increase in pH (from 7.46 to 8.69) occurred in the soil that was enriched with Na-bentonite (S+SB). After 14 months of the pot experiment, the water content was the highest in the S+SB+Fa (33.15%), although the pH of the S+P+Fa and S+SB+Fa ranged from 7.68 to 8.0. At the end (t_14_), both amendments had efficiently immobilized the bioavailable fractions of Zn (87–91%) and Cd (70–83%), whereas the efficacy of Pb binding was relatively low (33–50%). Significant differences (ANOVA, LSD, p≤0.05) were also observed in the pH and soil extractable content of Cd and Pb between S+Fa and CS. At t_14_ plant cultivation in S+Fa resulted in a decrease in the bioavailability of metals (about 10% for Cd and 18% for Pb) and pH (from 7.32 to 6.6) as compared to the CS.

### Grass biomass and the uptake of trace elements in plant tissue

**Fig 2:**
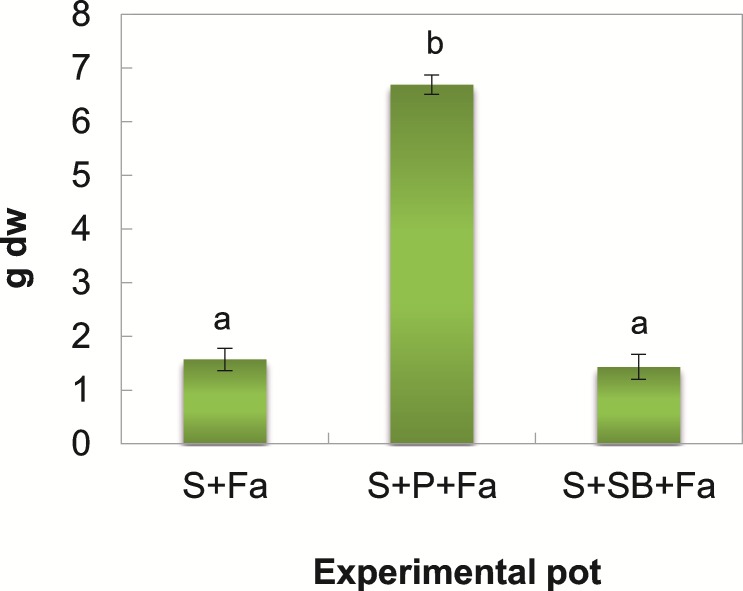
The plant biomass collected from the untreated and treated soils at t_14_. Means with the same letter(s) are not significant at p <0,05 within parameter between the untreated and treated soils.

The differences in the biomass of the plants harvested at t_14_ from the untreated and treated soils are shown in Fig 2. The highest plant biomass was determined in the S+P+Fa (6.69 g dw^-1^), whereas in the S+SB+Fa and the S+Fa, it was significantly lower and ranged from 1.43–1.57 g dw^-1^.

The harvested plants differed in the level of heavy metal accumulation in the roots and shoots (Table 4). The lowest Zn and Cd uptake in the shoots was determined in plants harvested from the S+P+Fa. Interestingly, Pb was under detection limit in the shoots for treatment with P. Meanwhile, the highest concentrations of Zn, Pb and Cd were observed in the shoots of plants grown in the presence of Na-bentonite (S+SB+Fa). These concentrations were 530,38, 11,93 and 4,15 mg kg^-1^ higher than in the shoots of plants collected from the S+P+Fa. The highest level of Pb and Cd were determined in the roots of plants cultivated in the S+SB+Fa and S+Fa. The addition of P resulted in a lower accumulation of Pb (2,3-fold) and Cd (2-fold) in the roots collected from the S+P+Fa than from the S+SB+Fa. The statistical analysis (ANOVA, LSD) showed no significant differences between the amounts of Cd in the roots that were obtained from all of the pots tested.

**Table 4:** The concentrations of heavy metals (mg kg^-1^ dw) in the plant shoots and roots at t_14_ (mean±SD; n = 3).

Treatments	Zn	Pb	Cd
**Shoots**			
S+Fa	770.27±10.71^**a**^	7.05±0.01^**a**^	5.49±0.46^**a**^
S+P+Fa	341.68±15.20^**b**^	nd	2.75±0.16^**b**^
S+SB+Fa	872.06±5.44^**c**^	11.93±0.88^**b**^	6.90±0.76^**c**^
**Roots**			
S+Fa	2010.10±197.05^**a**^	541.91±58.16^**a**^	354.97±11.49^**a**^
S+P+Fa	1597.60±272.50^**a**^	155.53±13.09^**b**^	108.79±1.11^**b**^
S+SB+Fa	2017.19±82.83^**a**^	349.37±37.62^**c**^	217.68±0.45^**c**^

Means with the same letter(s) are not significant at p <0,05 within each parameter between the untreated and treated soils at t_14_.

### Effects of amendments on microbial biomass and community structure

The obtained data indicated a substantial increase in the bacterial (BB) and fungal (FB) biomass over the course of the experiment (Table 5). Soil amendment with P increased BB by 51% and FB by 87% at t_0_ as compared to CS, although BB and FB in S+SB were not significantly altered (ANOVA, LSD). Consequently, at t_14_ BB and FB in the S+P+Fa were 3.5- and 7.7-fold higher as compared to CS, although at t_0_-t_14_ the highest increase in BB (6.6-fold) and FB (10-fold) in comparison with CS was observed for the SB treated soil over the course of the experiment. Independent of soil treatment, the contribution of microbial groups could be ordered as follows BG(-) > FB > BG(+) > AB. As H’ indicated (Table 5), the most diverse among the tested soils at t_0_-t_14_ were the S+P and S+P+Fa, while the other soils did not show significant modifications in any of the treatment periods.

**Table 5:** The content of PLFA markers and diversity index (Shannon H’) for the control, untreated and treated soils at t_0_, t_2_ and t_14_ (mean±SD; n = 3).

Sampling time	Treatments	TB	BB	FB	BG (+)	BG (-)	AB	H’
		(nmol g^-1^ dw)						
t_0_	CS	14.58±0.44^**a**^	12.53±0.02^**a**^	2.04±0.43^**a**^	6.39±0.39^**a**^	5.83±0.35^**a**^	3.06±0.26^**a**^	0.23±0.02^**a**^
	S+P	41.58±1.16^**b**^	25.69±1.83^**b**^	15.89±2.99^**b**^	12.24±0.88^**b**^	13.74±0.89^**b**^	3.77±0.31^**b**^	0.45±0.01^**b**^
	S+SB	13.47±0.55^**a**^	11.44±0.34^**a**^	2.03±0.21^**a**^	5.88±0.10^**a**^	5.41±0.17^**a**^	2.60±0.12^**a**^	0.26±0.04^**a**^
t_2_	CS	16.7±1.52^**a**^	14.34±1.30^**a**^	2.39±0.22^**a**^	6.65±0.60^**a**^	7.51±0.68^**a**^	2.91±0.26^**a**^	0.25±0.03^**a**^
	S+P	52.31±1.44^**b**^	33.07±1.69^**b**^	19.24±3.13^**b**^	12.20±0.48^**b**^	22.24±0.63^**b**^	4.13±1.03^**a.b**^	0.44±0.04^**b**^
	S+SB	34.80±0.40^**c**^	30.24±0.27^**b**^	4.56±0.14^**a**^	13.57±0.03^**c**^	16.81±0.53^**c**^	7.25±0.11^**c**^	0.26±0.02^**a**^
t_14_	CS	13.73±0.43^**a**^	9.96±0.10^**a**^	3.77±0.33^**a**^	4.68±0.29^**a**^	5.14±0.23^**a**^	1.95±0.03^**a**^	0.46±0.00^**a**^
	S+Fa	50.71±0.07^**b**^	41.17±0.21^**b**^	9.54±0.14^**b**^	19.76±0.11^**b**^	23.86±0.35^**b**^	10.16±0.31^**b**^	0.50±0.02^**a**^
	S+P+Fa	104.20±0.82^**c**^	65.68±1.09^**c**^	38.52±1.92^**c**^	27.00±0.50^**c**^	48.63±1.09^**c**^	9.52±0.13^**b**^	0.60±0.05^**b**^
	S+SB+Fa	56.10±0.40^**d**^	40.40±0.62^**b**^	15.70±0.22^**d**^	14.71±0.60^**d**^	30.04±0.54^**d**^	5.52±0.87^**c**^	0.52±0.01^**a**^

TB–total microbial biomass; BB–bacterial biomass; FB–fungal biomass; BG (+)–Gram-positive bacteria biomass; BG (-)—Gram-negative bacteria biomass; AB–actinomycetes biomass; H’–biodiversity index. Means with the same letter(s) are not significant at p <0,05 within each parameter between the control, untreated and treated soils at t_0_, t_2_ and t_14_.

**Fig 3:**
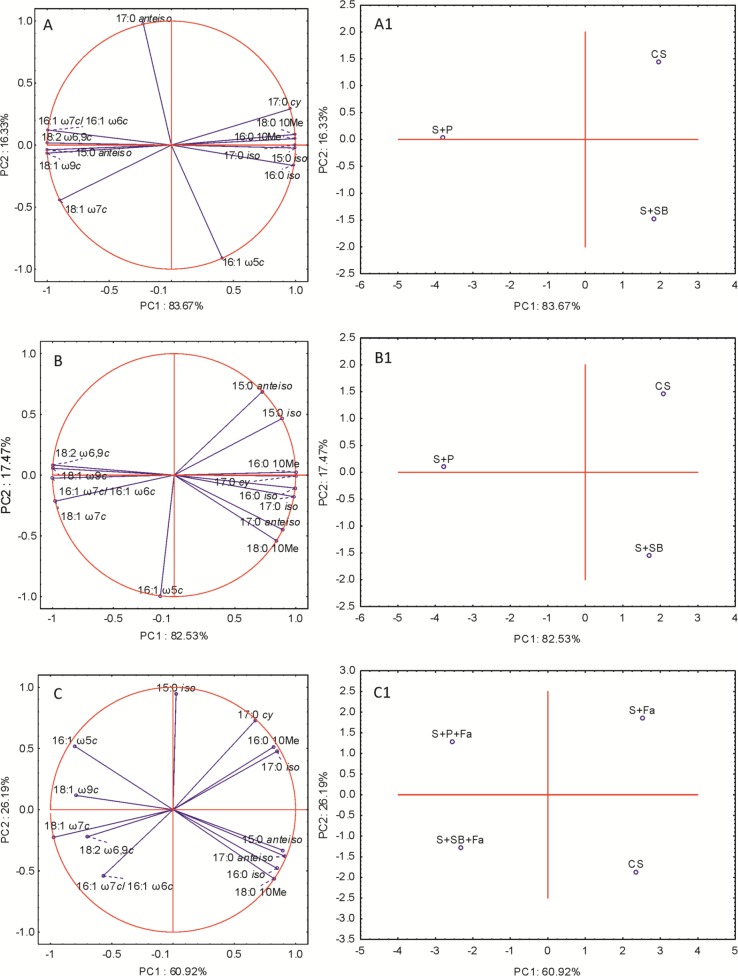
Projection of individual phospholipid fatty acids along PC1 and PC2 (A, B, C) and the PLFA profiles of the control, untreated and treated soils (A1, B1, C1) at t_0_ (A, A1), t_2_ (B, B1) and t_3_ (C, C1). The samples with similar PC1 and PC2 values are included into a cluster.

The PLFA data set was also subjected to the selection of a subset of significant fatty acids (ANOVA, p>0,05) to illustrate the differences and similarities between tested soils. Eleven high-loading PLFAs were entered into the principal component analysis (PCA), whereas 13:0 *anteiso*, 14:0 *iso*, 14:0 *anteiso*, 18:0 *iso* and 18:1 ω5*c* were not significant (ANOVA, p>0,05). At times t_0_-t_14_, the factor analysis provided two factors that explained 82.48–90.98% of the variance (Fig 3). Based on the analysis of PLFAs profiles, it was found that P and SB had an impact on the composition of phospholipid fatty acids in the treated soils. Those that most correlated with PC1 were saturated fatty acids: 16:0 10Me 16:0 *iso*, 17:0 *iso* and 17:0 *anteiso* and unsaturated fatty acids: 16:1 ω7*c*/16:1 ω6*c*, 18:1 ω7*c*, 18:1 ω9*c* and 18:2 ω6,9*c*, whereas 15:0 *iso*, 17:0 *anteiso*, 17:0 *cy* and 18:0 10Me and 16:1 ω5*c* were correlated with PC2 (Fig 3A–3C). Over the course of the experiment, the projections of the PLFA profiles changed along the PC1 and PC2 axes. At t_0_ and t_2_, the S+SB and CS formed one cluster for PC1 while the S+P was grouped independently (Fig 3A1 and 3B1). At t_14_ the S+P+Fa and S+SB+Fa formed one cluster, whereas CS and S+Fa grouped together along PC1 (Fig 3C1). Conversely, at t_0_ and t_2_ the S+P and CS formed one cluster, while the S+SB grouped separately along PC2 (Fig 3A1 and 3B1). At t_14_ the changes in PLFA profiles clearly indicated the different localizations of the tested soils along PC2. As is shown in Fig 3C1, while the CS and S+SB+Fa formed one cluster, the S+P+Fa and S+Fa did not tend to group along PC2. At t_14_ plant cultivation did not differentiate the PLFAs that were isolated from CS and S+Fa along the PC1 axis.

### Functional diversity of the soil microorganisms

The functional diversity of the soil microbial community (H’) and the substrate richness (S) indices for the soil microbiome after t_0_, t_2_ and t_14_ are presented in Table 6. In the treated S+P+Fa and S+SB+Fa at t_14_, H’ was similar to its initial values at t_0_. In addition, plant cultivation caused a two-fold increase of H’ for S+Fa as compared to CS at t_14_. Meanwhile, a similar trend in the value of S was observed for the S+P+Fa and S+SB+Fa. A decrease of AWCD at t_0_-t_2_ was observed for all of the tested soils. At t_14_ plant development significantly influenced the AWCD values for the S+P+Fa and S+SB+Fa as well as for the S+Fa.

**Table 6:** Physiological indicators AWCD, H’ and S for the control, untreated and treated soils at t_0_, t_2_ and t_14_ (mean±SD; n = 3).

Sampling time	Treatments	H’	S	AWCD
				(OUs)
t_0_	CS	1.28±0.01^**a**^	23.00±1.41^**a**^	449.13±2.88^**a**^
	S+P	1.41±0.01^**b**^	29.33±0.58^**b**^	783.52±2.10^**b**^
	S+SB	1.26±0.03^**a**^	20.00±2.00^**a**^	290.48±18.10^**c**^
t_2_	CS	0.96±0.05^**a**^	22.00±1.41^**a**^	163.07±12.90^**a**^
	S+P	1.33±0.05^**b**^	29.33±2.08^**b**^	469.29±33.10^**b**^
	S+SB	0.75±0.15^**c**^	3.00±0.50^**c**^	8.14±1.65^**c**^
t_14_	CS	0.47±0.01^**a**^	3.00±0.00^**a**^	17.35±2.34^**a**^
	S+Fa	0.98±0.08^**b**^	11.33±1.16^**b**^	114.25±7.82^**b**^
	S+P+Fa	1.43±0.01^**c**^	28.33±0.58^**c**^	741.91±27.75^**c**^
	S+SB+Fa	1.29±0.03^**d**^	21.50±2.00^**d**^	127.79±5.48^**b**^

OUs–omnilog units. Means with the same letter(s) are not significant at p <0,05 within each parameter between the control, untreated and treated soils at t_0_, t_2_ and t_14_.

**Fig 4:**
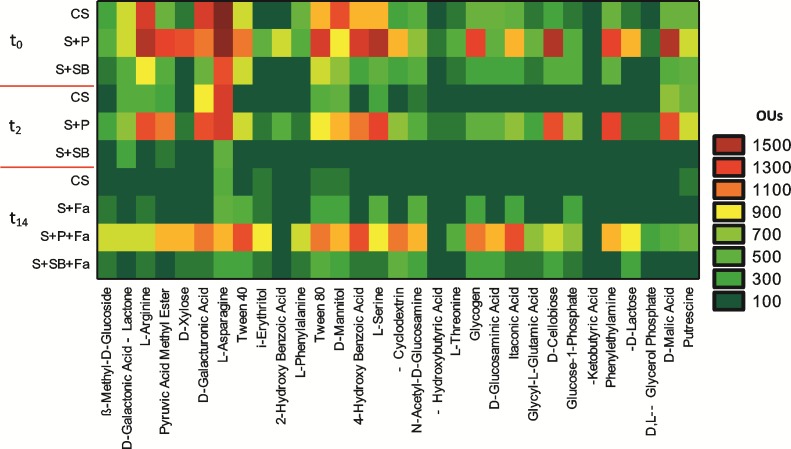
The activity of microorganisms in tested soils at t_0_—t_14_ (mean±SD; n = 3). The response of each compound was presented as a color scale ranging from dark green (0 OUs) to burgundy (1500 OUs).

The pattern of different the carbon sources that were utilized by the soil microbiome on EcoPlate^TM^ at t_0_-t_14_ is presented in Fig 4. The analyzed carbon substrates were divided into six main groups: carbohydrates, polymers, carboxylic acids, amino acids, amines/amides and surfactants. For the P and SB treatments, the soil microorganisms mainly used the following groups of carbon sources at t_0_ and t_14_: carbohydrates (D-mannitol, D-xylose and D-cellobiose), carboxylic acids (D-galacturonic acid, 4-hydroxybenzoic acid and D-malic acid) and amino acids (L-arginine, L-aspartate and L-serine). However, the data for t_2_ indicated a very low microbial activity in S+SB, which was manifested by the utilization of only three substrates (D-galactonic acid, γ-lactone, pyruvic acid methyl ester and L-asparagine). In comparison, in CS and S+Fa, the microorganisms mainly metabolized two carbohydrates (i-erythritol and D-mannitol) and two amino acids (L-asparagine and L-phenylalanine). The most significant difference in the physiological diversity between CS and S+Fa was revealed at t_14_, when the microorganisms metabolized 12 and 5 substrates in these soils, respectively.

### Soil enzymes activity

**Fig 5:**
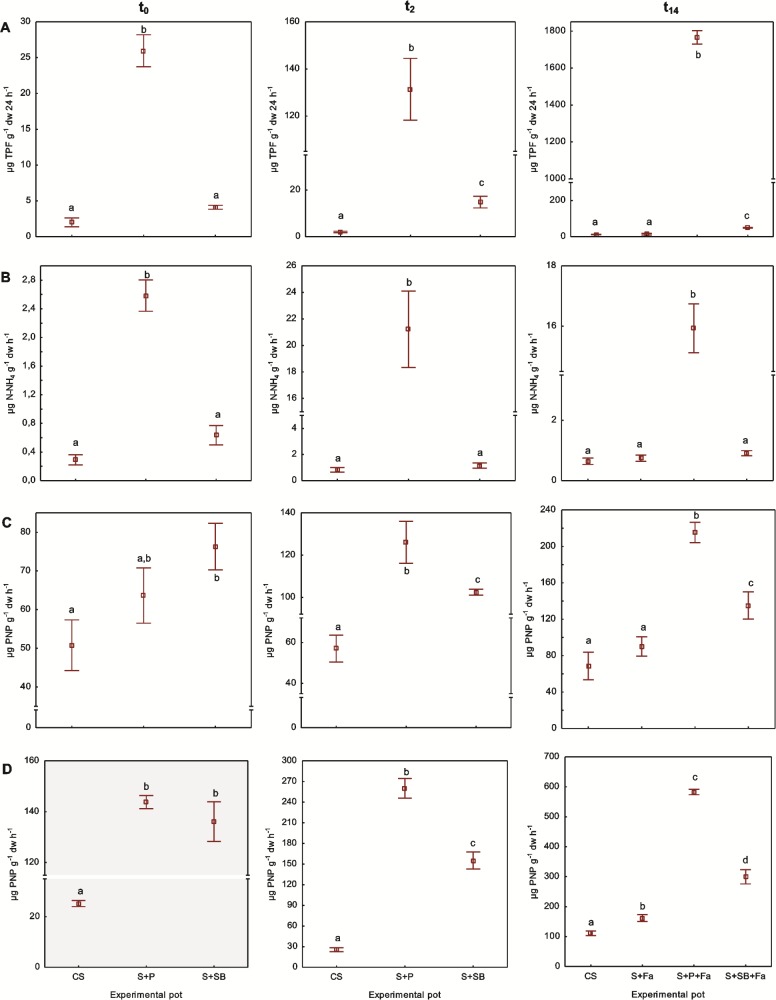
The activity of dehydrogenase (A), urease (B), acid phosphatase (C) and alkaline phosphatase (D) in the control, untreated and treated soils at t_0_–t_14_ (mean±SD; n = 3). Means with the same letter(s) are not significant at p <0,05 within tested parameter between the control, untreated and treated soils.

Dehydrogenases (Dha) activity data indicated a significant increase in the microbial metabolic activity due to the use of P as compared to SB. The Dha in the S+P at t_0_ exhibited a 13-fold higher activity than in the CS, whereas in the S+SB, it was 2.2-fold higher than in the CS. This trend generally remained constant over time. Finally, from t_0_ to t_14_ the P and BS amendment caused a 70- and 12-fold increase of Dha activity in the S+P+Fa and S+SB+Fa, respectively, as compared to its initial activity (t_0_). No significant changes in the Dha activity between the S+Fa and the CS were found at the end of the experiment (ANOVA, LSD, p≤0.05) (Fig 5A). The activity of Deh was positively correlated with BB (R^2^ = 0.61, p≤0,01) and FB (R^2^ = 0.72, p≤0,01).

Also, the application of P significantly influenced the ureases activity (U). Their initial activity in the S+P was 9-fold higher than in the CS and increased from 2.59 to 15.93 μg N-NH_4_ g^-1^ dw of soil during t_0_-t_14_. Over the course of the experiment, the lowest U activities were measured in the S+SB and CS. No significant changes between U activity in the S+SB+Fa, CS and S+Fa were found, even though its activity was higher in the SB treatment in comparison with the CS and S+Fa. The U activity was not affected by the growth of tall fescue in S+Fa in comparison with the CS (Fig 5B). The activity of U was positively correlated with FB (R^2^ = 0.64, p≤0,01).

Pulp and Na-bentonite amendment resulted in an increase in the acid phosphatase (Pac) activity from t_0_ to t_14._ The application of P and BS at t_0_ increased the Pac activity in the S+P and S+SB by 20 and 33%, respectively, as compared to the CS. At the end of the experiment, the Pac activity in the S+P+Fa was 2- to 3-fold higher in comparison with the S+SB+Fa, CS and S+Fa. The lowest activity urease was exhibited at t_14_ in the CS and S+Fa with no significant difference between these soils (Fig 5C). The activity of Pac was positively correlated with BB (R^2^ = 0.79, p≤0,01), FB (R^2^ = 0.75, p≤0,01) and BA (R^2^ = 0.53, p≤0,05) and negatively correlated with Zn (R^2^ = 0.81, p≤0,01) and Cd (R^2^ = 0.81, p≤0,01).

Alkaline phosphatase (Pal) activity was also modified by both soil amendments. At t_0_ after P and SB application, the Pal activity was 5.4–5.7-fold higher than in the CS, although no significant changes in its activity were found between the S+P and S+BS. At t_14_ Pal exhibited the highest activity in the S+P+Fa. It increased about 75% as compared to the initial activity (t_0_) and about 81% in comparison with its value in the CS. In comparison, Pal activity in S+BS+Fa increased by 45% over the whole experiment and about 3-fold as compared to the CS at the last sampling day. In all of the tested soils, Pal activity showed significant differences between the control and untreated soils, and was higher (46%) at t_14_ in the S+Fa than in the CS (Fig 5D). The activity of Pal was positively correlated with BB (R^2^ = 0,79, p≤0,01), AB (R^2^ = 0.53, p≤0,05) and FB (R^2^ = 0.81, p≤0,01) and negatively correlated with Zn (R^2^ = 0.74, p≤0,01) and Cd (R^2^ = 0.72, p≤0,01).

### Soil indices and cluster analysis

The values of M_W_ (soil fertility index), SQI (soil quality index) and SAI (soil alteration index) calculated for all of the tested soils are presented in Table 7. The values of M_w_ and SQI showed an 11- and 5-fold increase for the soils that were amended with P at t_0_-t_14_, while at those times the SAI increased the most (10-fold) for the SB treatment. A greater improvement of soil functioning was observed in the S+P+Fa at t_14_ than in the S+SB+Fa. At the last sampling day, the highest values of M_W_, SQI and SAI were calculated for the S+P+Fa and they were 33-, 7- and 10-fold higher, respectively, as compared to their values for the CS. In comparison, plant development had a relatively minor impact on the soil indices. The values of M_w_, SQI and SAI for S+Fa were only about 2-, 2- and 3-fold higher than for the CS, respectively.

**Table 7:** Soil fertility (M_W_), quality (SQI) and alteration (SAI) indexes for the control, untreated and treated soils at t_0_, t_2_ and t_14_ (mean±SD; n = 3).

Sampling time	Treatments	M_W_*	SQI	SAI
t_0_	CS	0.00^**a**^	0.00^**a**^	16.26^**a**^
	S+P	0.09^**b**^	0.19^**b**^	75.53^**b**^
	S+SB	0.05^**c**^	0.21^**b**^	13.33^**a**^
t_2_	CS	0.00^**a**^	0.04^**a**^	14.86^**a**^
	S+P	0.21^**b**^	0.53^**b**^	100.71^**b**^
	S+SB	0.06^**c**^	0.40^**c**^	53.15^**c**^
t_14_	CS	0.03^**a**^	0.14^**a**^	23.05^**a**^
	S+Fa	0.05^**b**^	0.25^**b**^	71.60^**b**^
	S+P+Fa	1.00^**c**^	1.00^**c**^	227.08^**c**^
	S+SB+Fa	0.12^**d**^	0.60^**d**^	137.98^**d**^

Means with the same letter(s) are not significant at p <0,05 within each parameter between the control, untreated and treated soils and at t_0_, t_2_ and t_14_.

*M_W_−organic matter content was used.

**Fig 6:**
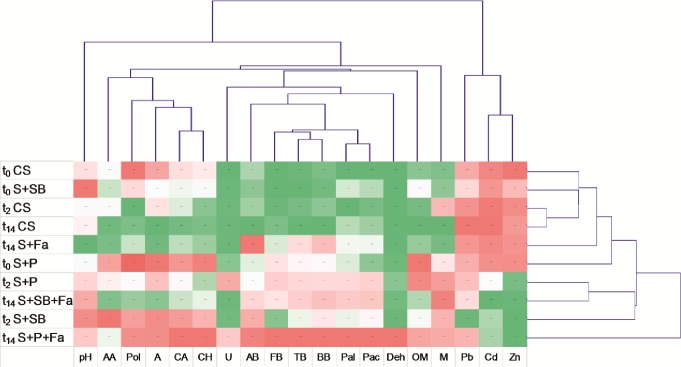
Cluster display of physicochemical and biological parameters of the control, untreated and treated soils at t_0_, t_2_ and t_14_ (mean±SD; n = 3). Legend: Zn–bioavailable fraction of Zn; Pb–bioavailable fraction of Pb; Cd–bioavailable fraction of Cd; M–moisture; OM–organic matter; Deh–dehydrogenase activity; Pac–acid phosphatase activity; Pal–alkaline phosphatase activity; U–urease activity; AA–amines/amides; Pol–polymers; A–amino acids; CA–carboxylic acids; CH–carbohydrates; BB–bacterial biomass; BA–actinomycetes biomass; FB–fungal biomass; TB–total biomass.

The similarity clustering of the different sampling times based on physicochemical and microbiological data is represented in Fig 6. The analysis showed two main separate clusters. One cluster included all of the biological parameters as well as pH, organic matter and moisture, whereas the concentrations of the bioavailable fractions of Zn, Cd and Pb were grouped in the second cluster. Similarly, clustering analysis of the studied soils based on the soil additives and plant development at t_0_, t_2_ and t_14_ demonstrated two separate clusters. In the first cluster, two subgroups were distinguished and grouped together. The first subgroup included the CS and S+Fa at t_0_-t_14_ and the S+SB and S+P at t_0_ with a strong similarity, while in the second subgroup the S+P at t_2_ and S+SB+Fa were the most similar to each other at t_14_ and the S+SB at t_2_. In turn, the S+P+Fa (t_14_), which was mostly based on the bioavailability of Zn and Cd as well as the microbial biomass and soil enzyme activities, were grouped independently.

## Discussion

Many environmental factors affect the bioavailability, toxicity and leachability of heavy metals in soil. However, relationships between soil physicochemical, microbiological parameters and trace elements contamination are not very often investigated under an aided phytostabilization [1,35,36]. For this reason, in this study the complex analyses of the physicochemical and biological properties of heavy-metal polluted soil were performed.

Depending on origin and composition of heavy metals stabilizers, organic and inorganic amendments can have variable effects on soil physicochemical properties. It was found in this work that pulp addition increased the moisture, organic matter content and pH of the soil, while Na-bentonite increased the water content and pH. These results were quite similar to those obtained using compost, leonardite and manure [15,37,38]. On the other hand, they are not in full agreement with results reported by Cheng and Hseu [39] and Hattab-Hambli at al. [40], who did not observe changes in the soil pH after the application of inorganic zeolite, bentonite and dolomite into soil.

With the incorporation of selected materials into the soil, the content of the bioavailable fraction of Zn, Cd and Pb significantly decreased as compared to the control soil. Both stabilizers reduced the available Zn and Pb at a similar level, however the bioavailable fraction of Cd was mainly immobilized in the soil with Na-bentonite. Likewise, the high efficiency of Zn, Cd and Pb immobilization by fly ash and red mud during an aided phytostabilization was established by Lopareva-Pohu et al. [41] and Pavel et al. [9]. The other materials that have proved to be efficient stabilizers of Pb, Cd and Zn in the soil from mining areas are green compost and compost with a high content of humic acids [15,42,43].

The toxicity symptoms in the planted control soil could possibly resulted from the combination of low pH, moisture, organic matter content and high CaCl_2_-extractable Zn, Pb and Cd fractions. In this study, the pulp promoted plant growth what was observed in a significant increase in the plant biomass, although the observed increment was smaller and comparable with the plant biomass collected from the untreated soil in the presence of Na-bentonite. Such a difference may have resulted from the nature of the materials used. The organic pulp could promote vegetation cover by providing K, N and P, which are essential for plant growth, thus raising the pH and chelating toxic metals slightly [44], while Na-bentonite in addition to binding metals could ensure good water conditions. Higher trace element concentrations were confirmed in the plant roots than in their aerial parts. The uptake of trace metals by plants significantly depended on organic and inorganic amendments in the soil. A greater decline of Cd and Pb content in the roots and Zn, Cd and Pb in the plant shoots grown in the presence of pulp was observed in comparison with the plants that were cultivated in the contaminated but untreated soil. This could be connected with a high affinity of Cd, Zn and Pb to organic matter and their ability to form stable metallo-organic complexes thereby reducing their bioavailability for plants [45]. Moreover, Rizzi et al. [46] and Nwachukwu and Pulfor [47] showed a lower accumulation of Zn and Pb in the grass roots of *Lolium italicum*, *F*. *arundinacea* and *L*. *perenne* that were collected from soils that were treated with an organic amendment as compared to the roots of the control. In contrast, a very high level of the accumulation of Cd, Zn and Pb in the plants that were harvested from the control soil could be a consequence of soil acidification that is caused by an increase in the secretion of different root exudates [48–50]. In comparison, Na-bentonite caused slightly higher amounts of trace elements in the aerial parts of plants that those obtained from the soil that was enriched with the pulp and the untreated soil. Thus, high soil pH could promote an increased uptake of metals from soil by plants [45].

As indicated our results, both soil amendments generally ameliorated the tested habitat and had a significant impact on microbial biomass, structural and functional diversity of microorganisms as well as soil enzymes activity. As PCA shown, the pulp and Na-bentonite distinctly separated the treated soils from the untreated ones. At the same time, it was established that the increase in the microbial biomass was greater in the soil with the pulp than in the soil treated with Na-bentonite. Despite this, Gram(-) bacteria dominated in all studied soils. Regardless of the soil treatment, the contribution of microbiological groups during the experiment was ordered as follows: BG(-) > FB > BG(+) > AB. The positive effects of other soil amendments such as green compost and cow manure on the biomass of soil microorganisms have been revealed [51,52], whereas the addition of charcoal into the soil decreased the microbial biomass [53]. In contrast to our results, the dominant group of bacteria in the soil that was enriched with manure, lime and bentonite under an aided phytostabilization with *Triticum aestivum* L. and *Salix alba* L. constituted G(+) bacteria. Surprisingly, in another study, Zornoza et al. [54] ascertained a higher total microbial biomass in the control soil than in the soil from a mining area that had been treated with manure and vegetated with *Pinus halepensis* Mill. and *Quercus rotundifolia* Lam. Additionally, they reported a very similar contribution of G(+) and G(-) bacteria in the remediated soil. Although the effects of organic and inorganic amendments on microbial biomass and diversity have been studied [51,53,54], their effects on composition of soil microbial communities and its functioning is not well documented.

In this experiment the high functional diversity of microorganisms in the amended soils was accompanied by a reduced availability of trace elements. The most active microorganisms were found in the soil treated with the pulp as was evidenced by the high activities of dehydrogenases, ureases, acid and alkaline phosphatases. The important role of plant cultivation on soil enzymes activity and microbial diversity has been confirmed in many studies [51,54,55]. The positive effects of organic waste and fermented sugar beet on soil biodiversity as well as the activities of dehydrogenase and acid phosphatase were observed by Bending et al. [55] and Kohler et al. [56]. The studies conducted by Mingorance et al. [57] and Li et al. [42] also revealed the positive influence of compost and *Lycopersicon esculentum* (Mill.), *Lolium perenne* L., *Pachyrhizus ahipa* (Wedd.) [57] and *Triticum aestivum* L. [42] on the activities of dehydrogenase and urease in soil under an aided phytostabilization. Similar to what was demonstrated in this study, Ascher et al. [58] proved an increase in the activities of acid phosphatase, alkaline phosphatase and urease in soil with zero-valent iron or beringite and *Lactuca sativa* L.(cv. Divina) vegetation. In our experiment, the enrichment of the soil with pulp resulted in higher values of the AWCD, H’ and S indices as compared to the soil that was amended with Na-bentonite. In the work of Galende et al. [59], poultry manure had a better influence on soil metabolic activity than other manures as was reflected by significantly higher values of the AWCD, H’ and S indices. On the other hand, Garcia-Sánchez et al. [60] did not observe any changes in the values of H' and metabolic richness (S) for Pb-, Ag- and Zn-contaminated soil in the presence of digestate and fly ash. Our statistical analysis indicated a strong positive correlation between acid and alkaline phosphatase activities with the bacterial and fungal biomass (p≤0.01) and decreasing concentrations of Zn and Cd (p≤0.05) in the treated soils. Such relationships could be related to higher pH and water content in the treated soils as compared to the corresponding values in the untreated and control soils that resulted from the high initial water content in the pulp and the high water-binding capacity of Na-bentonite.

There are no available reports concerning the effect of soil additives on soil indices (M_W_, SQI and SAI) during the remediation by an aided phytostabilization of soil that has been polluted with trace elements. Only a lack of a correlation between a reduction of Cd mobility in soil and its fertility [32] and the increase of the SAI value in the aftermath of restored vegetation cover and soil fertilization on mining area [33,34] have been earlier observed. According to Erkossa et al. [61] and Paz-Ferreiro et al. [62], organic amendments generally improved quality of soil contaminated with heavy metals. In our study the application of pulp into the mine-smelter soil clearly increased the rate of soil fertility (M_W_), its quality (SQI) as well as its dynamic changes (SAI) compared to other soils that have been studied.

## Conclusions

This study demonstrated that the use of pulp and Na-bentonite as well as the grass *Festuca arundinacea* Schreb. cultivar Asterix appeared to be a good options to reduce a high concentration of Zn, Cd and Pb in contaminated soil and improve conditions for plant growth. Although the application amendments increased the soil enzymes activity and diversity of microorganisms as well as plant biomass, these measurement values were generally greater for pulp than Na-bentonite treatment. The pulp reduced the bioavailability of trace metals, had apparent positive effects on soil quality essential for plant growth and microbial functioning, therefore it can be considered to be the most promising candidate material for use in aided phytostabilization. On the other hand, Na-bentonite proved mainly to be more effective in decreasing the heavy metal concentrations in the soil in comparison with the pulp. In conclusion, our comprehensive approach facilitated to determine the effect of the amendments application on soil and microbial parameters and understand this old management practice on soil quality.
